# In this month’s Bulletin

**DOI:** 10.2471/BLT.25.001225

**Published:** 2025-12-01

In the editorial section, Sarah L Barber (754) commemorates three decades of public health policy work by the WHO Kobe Centre.

## Nigeria

### Neglected tropical diseases 

Uchechukwu Madukaku Chukwuocha et al. (757–765) describe the use of photographs for surveillance.

## Region of the Americas

### Treating snakebite

Clara Guerra-Duarte et al. (785–798) review the evidence for use of antivenom. 

## Global 

### Input to health guidelines and other normative products

Meghan A Bohren et al. (766–784) explore community engagement methods.

### Increased caesarean births

Rana Islamiah Zahroh et al. (799–806) detail possible policy options.

### Paediatric azithromycin and nitrofurantoin

Yasir Bin Nisar et al. (807–813) present target product profiles.

### Workforce, regulation and capacity improvements

Vivian Lin et al. (814–822) explore requirements for traditional medicine integration.

### Achieving the 2030 Immunization Agenda

Mickey Chopra et al. (823–825) examine potential impacts of digital transformation.

### Artificial intelligence for diagnostic tests

Jicui Dong et al. (826–828) augment local production.

